# Sociotechnical Factors Affecting Patients’ Adoption of Mobile Health Tools: Systematic Literature Review and Narrative Synthesis

**DOI:** 10.2196/36284

**Published:** 2022-05-05

**Authors:** Christine Jacob, Emre Sezgin, Antonio Sanchez-Vazquez, Chris Ivory

**Affiliations:** 1 University of Applied Sciences Northwestern Switzerland Olten Switzerland; 2 The Abigail Wexner Research Institute at Nationwide Children's Hospital Columbus, OH United States; 3 NORC at the University of Chicago Chicago, IL United States; 4 Innovative Management Practice Research Centre Anglia Ruskin University Cambridge United Kingdom

**Keywords:** telemedicine, smartphone, mobile phone, electronic health record, public health practice, technology, perception, health education, mHealth, mobile health, telehealth, eHealth, patients

## Abstract

**Background:**

Mobile health (mHealth) tools have emerged as a promising health care technology that may contribute to cost savings, better access to care, and enhanced clinical outcomes; however, it is important to ensure their acceptance and adoption to harness this potential. Patient adoption has been recognized as a key challenge that requires further exploration.

**Objective:**

The aim of this review was to systematically investigate the literature to understand the factors affecting patients’ adoption of mHealth tools by considering sociotechnical factors (from technical, social, and health perspectives).

**Methods:**

A structured search was completed following the participants, intervention, comparators, and outcomes framework. We searched the MEDLINE, PubMed, Cochrane Library, and SAGE databases for studies published between January 2011 and July 2021 in the English language, yielding 5873 results, of which 147 studies met the inclusion criteria. The PRISMA (Preferred Reporting Items for Systematic Reviews and Meta-Analyses) guidelines and the Cochrane Handbook were followed to ensure a systematic process. Extracted data were analyzed using NVivo (QSR International), with thematic analysis and narrative synthesis of emergent themes.

**Results:**

The technical factors affecting patients’ adoption of mHealth tools were categorized into six key themes, which in turn were divided into 20 subthemes: usefulness, ease of use, data-related, monetary factors, technical issues, and user experience. Health-related factors were categorized into six key themes: the disease or health condition, the care team’s role, health consciousness and literacy, health behavior, relation to other therapies, integration into patient journey, and the patients’ insurance status. Social and personal factors were divided into three key clusters: demographic factors, personal characteristics, and social and cultural aspects; these were divided into 19 subthemes, highlighting the importance of considering these factors when addressing potential barriers to mHealth adoption and how to overcome them.

**Conclusions:**

This review builds on the growing body of research that investigates patients’ adoption of mHealth services and highlights the complexity of the factors affecting adoption, including personal, social, technical, organizational, and health care aspects. We recommend a more patient-centered approach by ensuring the tools’ fit into the overall patient journey and treatment plan, emphasizing inclusive design, and warranting comprehensive patient education and support. Moreover, empowering and mobilizing clinicians and care teams, addressing ethical data management issues, and focusing on health care policies may facilitate adoption.

## Introduction

Mobile health (mHealth) tools have emerged as a promising health care technology that may contribute to better access to health services, enhanced quality of care, and cost savings [1-6]. These novel technologies may also present an opportunity to enhance communication between patients and their health care providers and facilitate self-monitoring and self-management [7-9], leading to better treatment outcomes. Patients’ adoption is a key factor for mHealth success; however, it has been recognized as one of the key challenges.

Results from several trials showed that up to 70% of patients who were invited to use mHealth technologies either declined to participate or dropped using the tools prematurely [10]. Trials that reported higher retention rates were usually conducted over a short time frame and may not necessarily reflect the situation in real-world adoption [11]. A survey study on the topic stated that >50% of the surveyed clinicians cited patient resistance as one of the key barriers to adoption [12]. Furthermore, several studies have established that only a small fraction of patients kept using mHealth tools in the long term, and that up to 80% of users would only show minimal engagement, using the tools <2 times [13,14]. Another study conducted on a large real-world cohort of 189,770 people reported that only 2.58% of the people who downloaded the app sustained its active use, concluding that the impact of such apps may remain minimal if they fail to reach the patients who need them most [15].

The scope of this study is to build a better understanding of the different factors that may affect patients’ adoption of mHealth technologies. This study defines mHealth as “medical and public health practice supported by mobile devices, such as mobile phones, patient monitoring devices, Personal Digital Assistants (PDAs), and other wireless devices” as per the World Health Organization’s Global Observatory of eHealth, which considers mHealth a subcategory of eHealth. Telemedicine is, in turn, a subcategory of mHealth and defined as “the communication or consultation between health professionals about patients using voice, text, data, imaging, or video functions of a mobile device. But it can be applied to other situations; the management of chronic diseases of patients living at home being one example” [16].

Accordingly, a systematic review was conducted to provide a precise and up-to-date description of factors that affect patients’ adoption of mHealth tools from a technology, social, and health perspective. It also reflects on potential implications and suggests directions for relevant stakeholders to overcome barriers to adoption and thus facilitate the use of mHealth by a broader population. This work is part of an ongoing research project that explores the clinicians’ perspective and supplements its initial findings, which have already been published [17].

Findings from this study will help inform health care professionals, technology providers, and policy makers by presenting them with an up-to-date and comprehensive review of key factors affecting patients’ adoption of mHealth tools, as reported in the academic literature. This can guide them in making more informed decisions to promote adoption and harness the potential advantages of these tools.

## Methods

### Overview

The methods for this review were drawn from the PRISMA (Preferred Reporting Items for Systematic Reviews and Meta-Analyses) guidelines [18] and the Cochrane Handbook [19], both of which provide guidance toward a rigorous and reliable literature review methodology. The review methods were defined in advance and the protocol was published in the PROSPERO (International Prospective Register of Systematic Reviews) and is available on the web [20]. The analysis did not require any major divergence from the initial protocol. The research question that guided this review was the following: “According to the literature, what are the social, technical and health factors impacting patients’ adoption of mHealth tools?”

### Search Strategy

A search of MEDLINE, PubMed, Cochrane Library, and SAGE databases in July 2021 identified the relevant studies. The scope of this review was narrowed to studies published in English between January 2011 and July 2021. Only original, peer-reviewed, and published papers were included in this study. Other forms, such as editorials, unsystematic reviews, interviews, comments, unstructured observations, and position papers, were excluded. We decided not to include articles on the basis of manual searches of reference lists for causes summarized in the Cochrane Handbook: “positive studies are more likely to be cited” and “retrieving literature by scanning reference lists may thus produce a biased sample of studies” [19].

**Figure 1:**
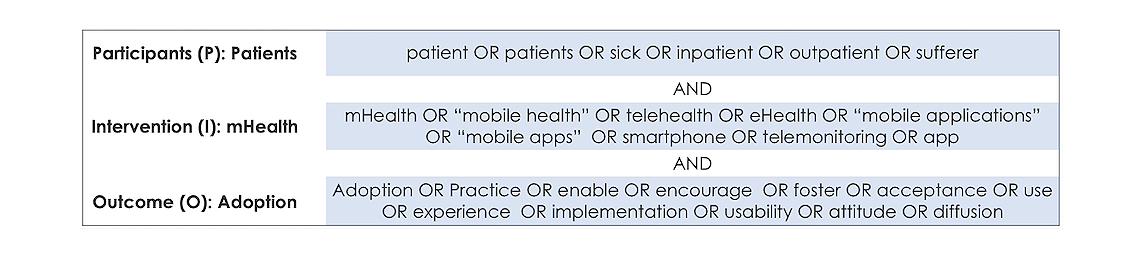
The search string according to the participants, intervention, comparator, and outcome (PICO) framework. mHealth: mobile health.

The search string shown in Figure 1 was developed according to the participants, intervention, comparators, and outcome framework [21]. There were no limitations to the types of conditions that qualified for inclusion, and both qualitative and quantitative studies were included. Comparators were not applicable to this study. Participants included studies that focused on patients. Interventions (mHealth) included studies involving smart device use such as mHealth apps or telehealth. Outcomes (adoption) included studies addressing the factors affecting mHealth technology adoption or use.

### Study Selection

Two researchers (CJ and ES) were involved in the screening, eligibility, and inclusion phases, and any divergence was agreed upon in the discussion between them. In cases in which they could not reach an agreement, a third reviewer (ASV or CI) discussed it with them and made the final decision. The research team used the open-source app Rayyan QCRI (Qatar Computing Research Institute) to facilitate collaborative screening [22]. Screening lasted from June to September 2021.

The inclusion and exclusion criteria, detailed in Textbox 1, were developed according to the participants, intervention, comparators, and outcome framework. Studies were excluded if they did not involve the use of mHealth or smart devices; focused solely on, for example, clinicians, caregivers, or technology providers; did not include patients; were not peer-reviewed; were editorials, interviews, comments, unstructured observations, or position papers; did not address the factors affecting adoption; or if the full text was not available, freely available, or available in English.

**Inclusion and exclusion criteria according to the PICO (participants, intervention, comparator, and outcome) framework.**

**Inclusion and exclusion criteria**

**Population**

- Include: focused on patients
- Exclude: focused only on clinicians, caregivers, or technology providers

**Intervention**

- Include: focused on solutions involving a smart device (eg, mobile health [mHealth] apps and telehealth)
- Exclude: using other technologies (eg, virtual reality and machine learning)

**Comparators**

- Does not apply

**Outcome**

- Include: addresses factors impacting patients’ adoption, acceptance, use, experience, usability, or attitude of using mHealth, regardless of the condition
- Exclude: focused only on mHealth success or development in general

**Publication type**

- Include: original, peer-reviewed, and published paper
- Exclude: editorials, interviews, comments, unstructured observations, and position papers

After completing the screening and resolving any conflicting views among the researchers, the selected full texts were assessed for eligibility independently by CJ and ES. Any disagreements were resolved through discussion with ASV or CI. The risk of bias was assessed using the Critical Appraisal Skills Program tool [23]. The checklist is included in Multimedia Appendix 1, and a Microsoft Excel sheet with the appraisal of the included studies can be accessed in Multimedia Appendix 2.

### Data Collection and Synthesis

The variety of procedures and results that were identified in the included studies was not homogeneous enough to enable a quantitative analysis of the data. Therefore, a narrative synthesis was used and structured around the social, health, and technical factors affecting patients’ adoption of mHealth solutions. NVivo (QSR International), a computer-assisted qualitative data analysis software, was used to assist with this task.

Data coding began with a preliminary data extraction grid that included themes based on previous research and technology acceptance frameworks; the initial codebook was informed by our previous work that aggregated the factors used in the most used frameworks [24]. More codes were added as they emerged during the review process. Thematic analysis by Braun and Clarke [25] was used to identify and extract themes under social, technical, and health factors addressed in the research question. Social factors include any social-related elements, such as the effects of people and groups influencing one another through culture; technical factors include elements related to the material sides of the technology, such as its ease of use and usability; and health-related factors were linked to elements such as the health condition itself and the patient’s health literacy. The phases of the thematic analysis are explained in detail in Multimedia Appendix 3. This process lasted from September to November 2021.

### Theoretical Framework

Health care technologies are generally more complex than tools that address individual user needs, as they usually support patients with comorbidities who are typically treated by multidisciplinary teams who might even work in different health care organizations. The special nature of how the health care sector operates and its high degree of regulation, normalized budget deficits, and the interdependence between health care organizations necessitate some crucial expansions to existing theoretical frameworks usually used when studying adoption.

Therefore, the authors were guided in their thinking about technology adoption by theoretical frameworks in the field of social studies of technology and sociotechnical theory; they view technology, roles, and practices and organizational structures as interacting parts of a mutually constituting ensemble of elements [26]. They used a consolidated model that the research team had previously published [24], in which they reviewed and aggregated the most used frameworks applied to technology adoption in health care. Most factors could be linked to one framework or another, but there was no single framework that could adequately cover all relevant and specific factors without some expansion. This led the authors to suggest a shift toward an extended framework that considers the complexity of the health care landscape, its highly regulated nature, and the interdependence between its different stakeholders [24]. This is aligned with what other scholars have also suggested, explaining that many of the broadly used frameworks adopt a technology-centered view focusing on the tool itself [27-30], and proposed a move to multidimensional models that go past usability to encompass the surrounding context, as well as societal and implementation challenges [27,28,30-33].

## Results

### Overview

**Figure 2:**
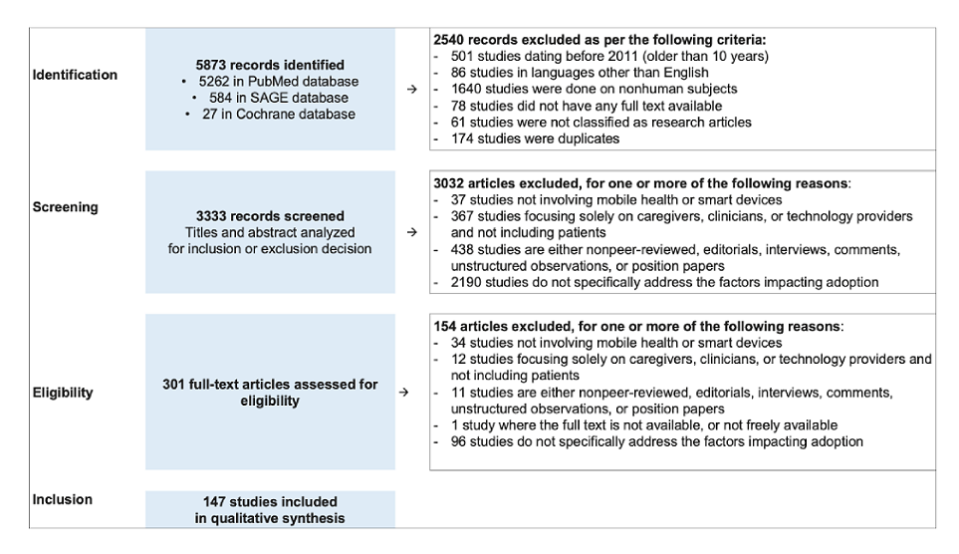
Study selection flow diagram on the basis of the PRISMA (Preferred Reporting Items for Systematic Review and Meta-Analysis) guidelines.

As shown in the study selection flow diagram (Figure 2), the search string yielded 5873 studies, of which 5262 (89.6%) were from PubMed, 584 (9.9%) from SAGE, and 27 (0.5%) from the Cochrane database. Of these 5873 studies, 2540 (43.2%) were excluded after limiting the scope to studies published in English and published after January 2011, leaving 3333 (56.8%) studies for screening. Screening of the titles and abstracts excluded another 3032 articles because 37 of them did not involve mHealth or smart devices; 367 focused solely on nonpatient populations such as clinicians, caregivers, or technology providers; 438 were editorials, interviews, comments, unstructured observations, position, or non–peer-reviewed papers; and 2190 did not address factors affecting adoption.

In the eligibility phase, 301 articles were included for full-text assessment. A total of 154 articles were excluded for the following reasons: 34 for not involving mHealth or smart devices; 12 for focusing solely on nonpatient populations such as clinicians, caregivers, or technology providers; 11 for being editorials, interviews, comments, unstructured observations, position, or non–peer-reviewed papers; 1 because the full text was not available; and 96 for not addressing the factors affecting adoption. This resulted in the inclusion of 147 articles for the qualitative synthesis [34-180].

### Characteristics of Included Studies

Multimedia Appendix 4 presents the sample characteristics of the included articles. Overall, 85 studies focused on patients, 24 on both healthy and sick people, 24 on patients and health care professionals, 4 on patients and caregivers, and 10 included patients and other populations, such as clinicians, researchers, policy makers, and medical students. From a disease area perspective, some were more represented than others in the included studies; 16 studies focused on diabetes and obesity, 13 on cardiovascular disease and heart failure, 13 on mental health, 11 on surgery, 10 on oncology, 9 on chronic diseases, 8 on primary care, and 6 on neurology and neurosurgery, whereas the other disease areas were represented ≤4 times in the included studies.

Most of the publications did not mention the use of a theoretical framework. Among those that used one, the Unified Theory of Acceptance and Use of Technology was the most common (n=12), followed by the Technology Acceptance Model (n=11) and the Diffusion of Innovation Theory (n=2). Other models were used only once, as described in Multimedia Appendix 4. From a geographical perspective: 46 studies were conducted in the United States, 12 in China, 10 in the United Kingdom, 8 in Canada, 5 in Australia, 5 in Germany, 5 in Singapore, whereas other geographies were covered in ≤4 studies. From a sample size perspective, most of the included studies had a sample size >100 participants (n=80), whereas most studies that included smaller samples were qualitative in nature and did not necessitate the larger samples that are typically required in quantitative approaches.

### Critical Appraisal

On the basis of the critical appraisal, 42.8% (63/147) studies did not clearly justify their choice of study design, but still used a design that is suitable for their objectives, 4.8% (7/147) did not report a clear participant recruitment strategy, 0.7% (1/147) did not provide sufficient details on the data collection techniques, 19% (28/147) did not report if the study procedure was reviewed for ethics approval, 18.4% (27/147) were not clear enough about their data analysis strategy and whether it was sufficiently rigorous, and 8.2% (12/147) did not sufficiently discuss the practical or policy implications of their findings. However, articles were not excluded based on technical quality to enable researchers to capture both theoretical and empirical contributions from the published studies.

### Social and Personal Factors

**Figure 3:**
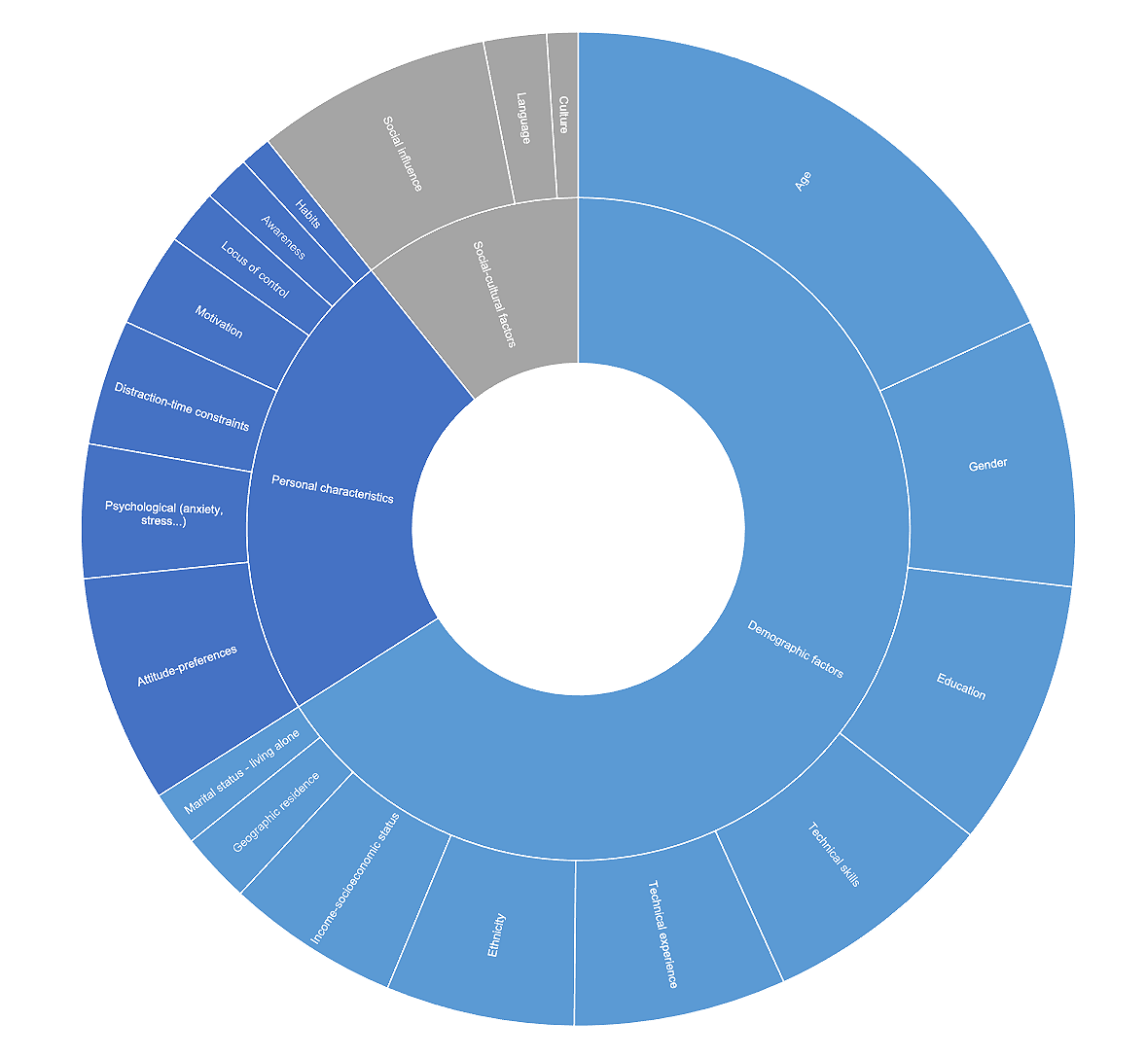
Overview of social and personal factors and their occurrence.

The social and personal factors affecting patients’ adoption of mHealth were categorized into three key themes: demographic factors, personal characteristics, and cultural and social elements. These were, in turn, subdivided into 19 subthemes. Figure 3 provides an overview of these social and personal factor themes and subthemes and their respective occurrence.

**Figure 4:**
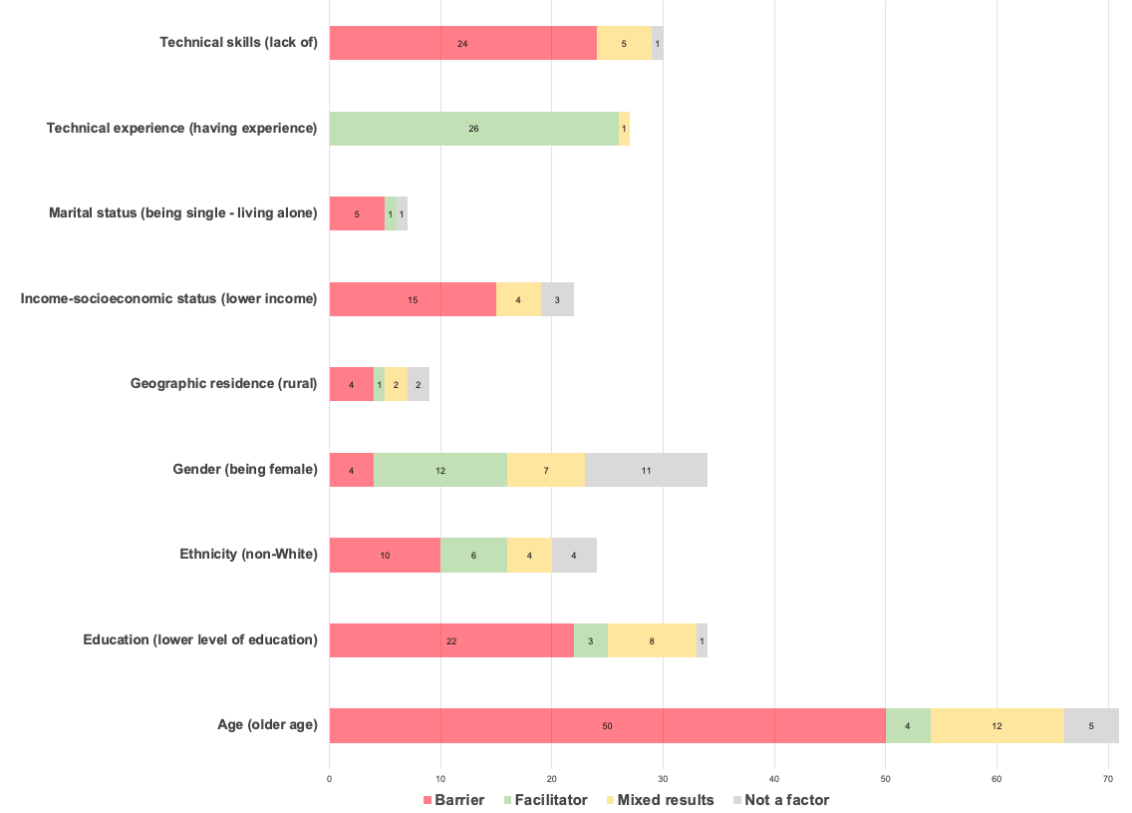
Subanalysis of the demographic factors according to their classification in the included studies.

Demographic factors were the most prominent, often related to matters such as age (n=71), gender (n=34), education (n=34), technology skills (n=30), technology experience (n=27), ethnicity (n=24), socioeconomic factors (n=22), geographic residence (n=9), and marital status (n=7). An in-depth analysis of the demographic factors was also done to clarify which factors were mostly cited as barriers (they hinder adoption), facilitators (they facilitate adoption), mixed results (their relationship to adoption is not linear and may vary based on context), or had no impact on adoption according to the included studies, this subanalysis is visualized in Figure 4.

Personal characteristics also played a central role, with factors such as patient attitudes and preferences (n=29), psychological factors (n=17), time constrain and distraction (n=16), and motivation (n=12) being in the center. Other personal characteristics were also mentioned, including the locus of control (n=7), awareness (n=6), and habits (n=5). These factors were complemented by cultural and social elements including social influence (n=30), language (n=8), and culture (n=4). Multimedia Appendix 5A details the social and personal factors affecting adoption, their occurrence, and the respective studies where they were identified.

### Technical and Material Factors

**Figure 5:**
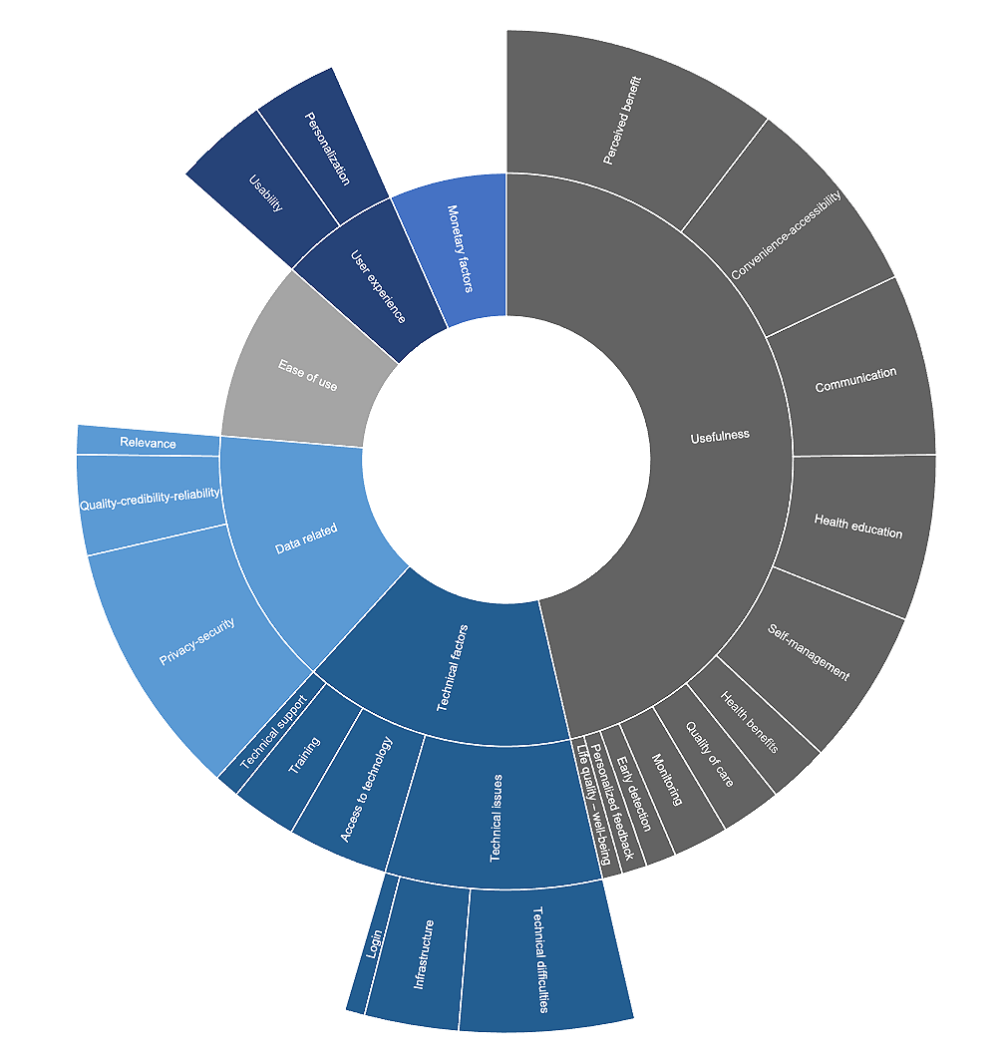
Overview of technical and material factors and their occurrence.

The technical and material factors affecting patients’ adoption of mHealth were categorized into six key themes, which were in turn divided into 20 subthemes: usefulness, ease of use, data-related, monetary factors, technical issues, and user experience. Figure 5 provides an overview of these technical and material themes and subthemes and their respective occurrence.

Usefulness was the most prominent factor in the technical and material clusters and was often related to matters such as perceived benefits and performance expectancy (n=55), convenience and accessibility (n=40), communication (n=36), health education (n=33), self-management (n=31), quality of care (n=12), health benefits (n=12), monitoring (n=11), early detection of symptoms (n=6), personalized feedback (n=5), and quality of life and well-being (n=4). Ease of use (n=54) was also very prevalent, as were data-related factors, mostly evolving around privacy and security (n=51), quality and credibility (n=20), and relevance (n=6).

There was also a frequent mention of monetary factors (n=35), such as cost and reimbursement, as well as user experience, where the focus was mostly on the usability of the tools (n=19) and personalization (n=17). Technical factors evolved around technical issues such as infrastructure and log-in problems (n=43), access to technology (n=20), training (n=13), and technical support (n=5). Multimedia Appendix 5B details the technical and material factors affecting adoption, their occurrence, and the respective studies where they were identified.

### Health-Related Factors

**Figure 6:**
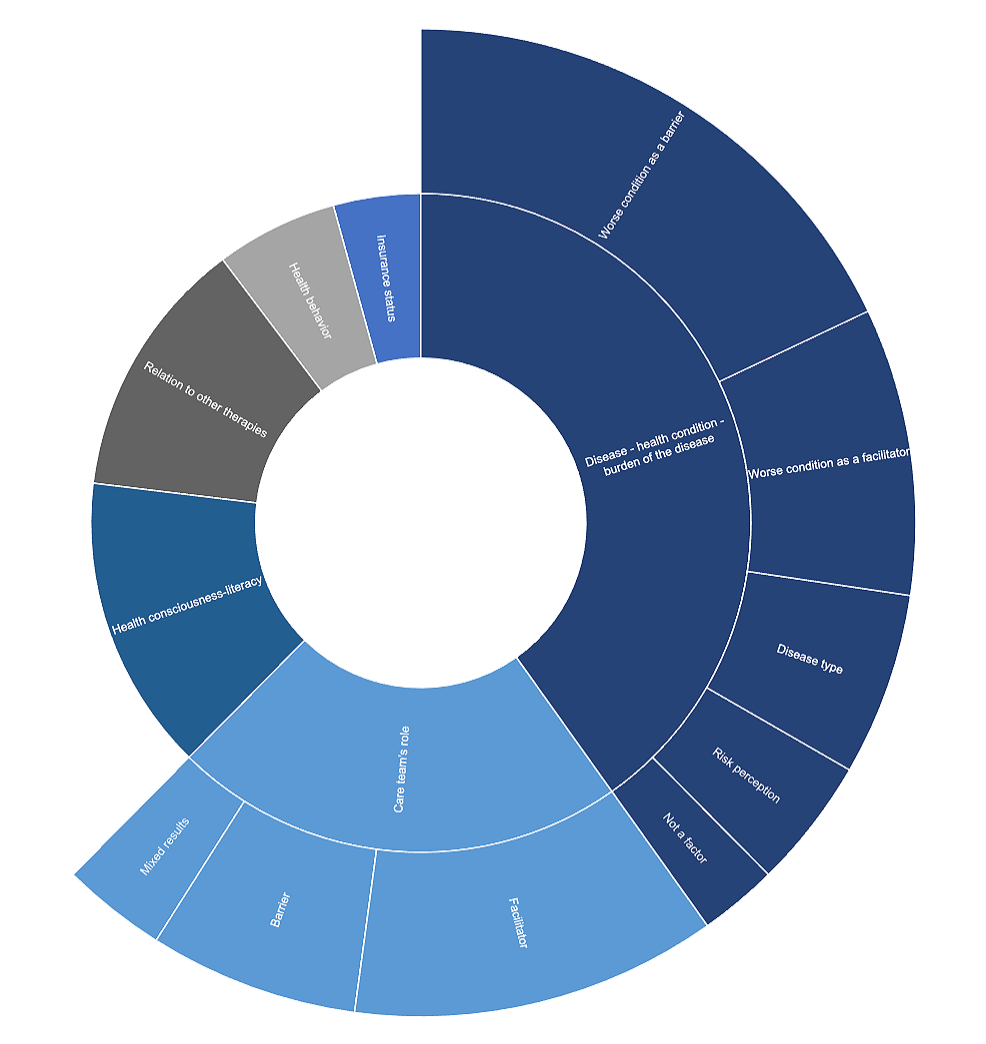
Overview of health-related factors and their occurrence.

Health-related factors affecting patients’ adoption of mHealth were categorized into six key themes: the disease or health condition, the care team’s role, health consciousness and literacy, health behavior, relation to other therapies and integration into patient journey, and the patients’ insurance status. Figure 6 provides an overview of these health-related themes and subthemes and their respective occurrences.

The disease or health condition that the patient had was not only the most prominent factor, often related to matters such as perceiving the worse condition as a barrier to adoption (n=21), but also a facilitator in other contexts (n=11). The disease type itself may also be a factor (n=7) and the patient’s risk perception of their health condition (n=5), whereas other studies found that the health condition was not a factor (n=3). Similarly, the care team’s role was mostly reported as a facilitator (n=14), but also sometimes as a barrier (n=8), although some papers reported mixed results (n=4).

Other health-related aspects such as health consciousness and literacy (n=17), relation to other therapies, and the integration of mHealth into the patient journey (n=15), as well as the patient’s baseline health behavior (n=7) and insurance status (n=5) were cited as potential factors that may affect health technology adoption. Multimedia Appendix 5C details the health factors affecting adoption, their occurrence, and the respective studies where they were identified.

### Principal Findings

The main findings of this review emphasize the central factors affecting patients’ adoption of mHealth tools. Analyzing the prevalence of the different factors sheds light on the significance of social and health-related factors that go beyond technical features, stressing their importance when developing and deploying these tools.

### Social and Personal Factors

The prominence of social and personal factors in the included studies highlights how mHealth adoption is closely connected to and shaped by the societal dynamics in which they are embedded. Demographic factors, personal characteristics, and other social and cultural elements may play a key role in patients’ willingness to adopt an mHealth tool.

### Demographic Factors

Age was the most prominent demographic factor, with older age mostly cited as a barrier, and many studies have reported a negative relationship between age and willingness to use such tools [34-55]. Some studies further explained that this may not be because of age per se but indirectly because of other factors such as older individuals facing physical or cognitive challenges [56,57], unfamiliarity with the use of technology or smartphones in general [58-63], or lack of phone ownership [64-66]. In the case of solutions dedicated to child patients, parents’ age was negatively associated with their willingness to use digital tools, whereas children’s age was positively associated with their willingness to adopt these tools [67].

Nevertheless, older age was cited as a facilitator in some studies, with older patients being among the highest adopters and the most adherent users [68-70], especially in cases where there is a clear need such as during the COVID-19 pandemic when a remote health service may help older patients minimize infection risk [71]. Other researchers have reported that age is not a factor, and that older patients are as interested as their younger counterparts, especially after adjusting for other factors, such as technology skills and experience [72-76]. This may explain why some studies concluded that increasing age should not necessarily be considered a limitation because it mostly depends on the context and other related factors [77-80], suggesting that ensuring ease of use and delivering better training could help close this gap [81-83], and that a better understanding of how the tools may help them improve their condition could motivate the adoption decision [84,85].

Gender was also a prominent demographic factor, with being female mostly cited as a facilitator, and many studies reporting on the positive relationship between being female and the willingness to use such tools [39,51,55,71,85-88], with some researchers describing that this may be because of gender-specific behavioral patterns, as women frequently undertook the role of *health care liaison* for their families [82], that mothers may experience more anxiety than fathers and are therefore more likely to seek alternative solutions [67]; therefore, these gender-related use patterns may very well be because of the care role that society assigns to women rather than gender per se [54,83]. Furthermore, this may be because of trial bias and self-selection bias presented by female participants, as seen in the patient characteristics of many mHealth studies. However, it is worth noting that an equally prevalent number of studies reported that gender was not significantly associated with the adoption decision [36,40,45,50,53,62,68,73,89-91].

Conversely, some studies have concluded that adoption is more widespread among male users [43,92,93], sometimes because of other related factors, such as more prevalent phone ownership among male members of a specific society [65]. Moreover, other researchers have established that gender is not necessarily a decisive factor, and that adoption may vary according to the context and other factors [49,94,95]. For instance, Abelson et al [77] explained that while women in their study were more likely than their male counterparts to be anxious about losing face-to-face communication with their care providers, they were also more likely to welcome the beneﬁt of avoiding unwarranted clinic visits. Other studies noted that women may tend to be more adopters of specific types of digital tools compared with others. For example, Beard et al [35] noted that women are more likely to adopt mental health apps, but less likely to adopt other types of apps that use entertainment, for example, compared with men. Gender-specific behavior may also differ according to the health condition in question, as reported by Foster et al [69], where adopters were most likely females in the depression trial and most likely males in the cardiovascular disease risk trial.

Education was another prominent demographic factor, with lower levels of education mostly cited as a barrier, and many studies have reported a positive relationship between the level of education and the willingness to use mHealth tools [35,49,53,55,64,65,72,84,92,96-98]. This was explained in some studies by lower access to, and skills in using technology [89,90], and lower eHealth literacy among the less educated in some contexts [44]. Only one study reported that education was not significantly correlated with adoption [68].

Unexpectedly, some studies concluded that lower education may, in some cases, facilitate adoption [45]. For example, people with less education may have higher health information needs that, in turn, foster their digital information–seeking behaviors and consequently promote adoption [88], or they may be more likely to seek alternative or supplementary solutions when care problems occur [99]. Other researchers established that education is not necessarily a conclusive factor, and that adoption may vary according to the context and other factors [95], such as lower rates of computer and internet access among the less educated [82], lower technical skills [78], and differences depending on the type of solution at hand [39,51]. Furthermore, Torrent-Sellens [83] found that the relationship between education and adoption was not linear but rather U-shaped, with usage being greater among participants with a secondary education and lower among those with primary and tertiary education.

Technology-related skills were predictably among the most prominent factors, with the lack of technology skills being cited as a barrier, and numerous studies have reported a positive relationship between technology skills and the willingness to use mHealth tools [52,62,74,80,100-103], especially among older patients who may lack these skills [61,69,77,79,103-105]. Some studies concluded that a lack of technical skills may be the underlying cause of lower adoption in older age groups, not their age as such [72]. However, one study stated that self-efficacy and a person’s perception of their own skills did not have an impact on adoption [36]. Although the lack of technical skills was typically perceived as a hindrance to adoption in the included articles, some studies reported that it is not necessarily the case; for example, if the person believes that everything can be learned, it is no longer considered a hindrance, meaning that the adoption decision also depends on the person’s attitude and openness to learn new skills [106] and on other contextual factors such as the availability of training and some additional help or support [58,64,66,107].

Similarly, technological experience was prominent, with all studies except one reporting a positive relationship between previous technology experience and adoption decisions, stating that factors such as previous smartphone use or ownership [84,92,96,107], ownership of wearable devices [49], use of health apps [38,46,54,56,83,97,109-113] or apps in general [40,48,78,85], and broad experience with digital technologies [102,114-118] may increase the odds of mHealth acceptance and adoption. However, Zhang et al [127] pointed out that even users with previous technology experience may still choose not to adopt a tool that they perceive as irrelevant or less useful compared with their traditional methods in receiving health care.

Ethnicity came up in several studies, with being non-White mostly reported as a barrier to adoption, and a negative relationship between mHealth acceptance and being of non-White ethnicity [35,71,84,119,120], sometimes relating the impact of ethnicity to other indirect factors, such as socioeconomic factors [62], lower health literacy [121], less access to technology [64,66], and insurance status [122]. However, some studies have reported mixed results [95], with ethnicity being a significant factor in some contexts and not a factor in others, as described by Foster et al [69], who highlighted that the relevance of ethnicity varied in the different trials they conducted depending on the health condition in question. Other researchers who also reported mixed results pointed out that ethnicity itself may not be the real factor, but rather other underlying triggers such as systemic racism and the existing disparities in health services that may have increased the need for such alternative solutions among non-White patients [123]. In other contexts, being non-White was reported as a facilitator of adoption [43,51,124,125], which was sometimes linked to other contextual factors such as non-White patients being less concerned about privacy issues compared with their White counterparts [77], or more use of mobile apps in general and the perception that mobile phones are essential, predominantly because of the lack of home landlines [44]. However, some studies concluded that ethnicity had no impact on patients’ decisions to adopt mHealth [50,53,86,90].

Although some researchers have established that socioeconomic factors such as income have no impact on mHealth acceptance [48,50,52], lower or insufficient income has often been reported as a barrier to adoption [37,43,44,57,63,77,83,84,90,91,126], frequently citing other interrelated factors such as insurance status, skills and education, or access to technology [51,82,89,127]. There are also studies that have reported mixed results where income may be a factor in some contexts but not in others [41,95], sometimes depending on other elements such as the level of education [62] or the specific health condition [39].

Geographic residence seems to also sometimes affect adoption, and several papers reported that living in a rural area is mostly considered a barrier to adoption [71,83], sometimes owing to lower technology access because of a less developed infrastructure in some rural areas [89]. Jaffe et al [50] reported that the prevalence of mHealth use in some regions more than others may also be related to other interrelated factors such as a lower number of COVID-19 infections in the regions that had lower adoption rates, most likely because there was less need for mHealth use in those regions with fewer cases. Rush et al [113] is the only study that concluded that living in a rural location may be a facilitator because of the long distances that a patient has to travel to access health care services and the life-saving effect that a remote service may have in such cases. Nevertheless, some studies have reported mixed results [76,95], whereas others have found that geographic residence was not a factor affecting adoption decisions [90,91].

Interestingly, marital status was also reported as a potential demographic factor affecting the adoption of these tools; seemingly, being single or living alone was usually a barrier [43,83,119], most likely because of the absence of accountability and support that a significant other may offer [82,95]. One study concluded that marital status has no impact on adoption [53], and another study reported that living alone or being single, divorced, or widowed may increase the odds of using mobile health [50].

### Personal Characteristics

Patients’ attitudes and preferences are among the most prevalent personal characteristics that may affect mHealth acceptance. For instance, preference for face-to-face interactions with their care team [37,77,115,119,127,128], resistance or openness to change [36,45,129,130], negative or positive perceptions of mHealth [41,43,56,60,62,83,87,102,114,131-134], lack of interest [55,58,69,101,103], and fear of technology [135] are all elements that play a role in whether a patient is more or less receptive to these technologies.

There are also important psychological aspects to consider, such as individual-level processes and meanings that influence mental states. For instance, although mHealth may increase the feeling of safety for some patients as they know they are being monitored or have access to additional safety measures [67,80], it may trigger a sense of anxiety and stress in others for many reasons, such as being constantly reminded of their symptoms and so their disease [80,101,107,118,136-139], with these feelings sometimes subsiding when patients become more comfortable managing their own condition [112]. Furthermore, sometimes patients may give up the use of mHealth because they are overwhelmed or struggling to cope with their condition [140], they do not want the additional stress of managing their condition and prefer relying on their care team [73,79], or they may lack the emotional capacity to even try to use the tools [69]. Interestingly, users may also abandon digital tools and choose face-to-face examinations because of their emotional need to have physical contact and get out of the house [115]. Conversely, mHealth may help overcome some psychological challenges by enabling patients to receive health care services in a more private way, particularly in stigmatized areas such as mental health services [85].

Distraction and time constraints may also interfere with mHealth adoption, some patients drop the apps because they tend to forget to use them [100,110,133], get too busy with other competing priorities that take up all their time [69,80,91,100,112,115,116,137,138,140,141], find the tools too time consuming [55], or get annoyed by the interference of the app with their daily life through frequent reminders at unsuitable times that cannot be customized to their schedules [118,120]. This factor may also relate to a patient’s existing habits [57] and how a successful adoption is tied to the person’s willingness to embed the mHealth tools in their day-to-day routines to become a natural part of their existing agendas [70,140]. Haldane et al [110] pointed out that it might be easier for newly diagnosed patients to adopt these tools compared with established patients who have been managing their conditions using traditional methods for a long time. Conversely, other scholars have concluded that habit may not necessarily be a hindrance to adoption in the case of user-friendly tools that only require minimal effort and no major change in the user’s daily habits and routines [142].

Motivation is another personal characteristic that emerged as a noteworthy determining factor of behavioral intention to use new health technologies [140,142,143]; hence, motivating patients to use mHealth may be a challenge, especially if they perceive the tools as a burden or as not useful to them [130,144]. The lack of motivation in general [100,107,145] or lack of engaging mechanisms within the apps themselves may also be a challenge to adoption [146], whereas apps that include motivational elements such as rewards or interactivity may encourage adoption [101,114,147]. Similarly, self-efficacy and locus of control may also affect patients’ decisions to adopt mHealth; people who feel that they are more in control of their life and their condition are more intrinsically motivated to adopt self-management tools [80,140,148], and they are more likely to adhere to the tools when they feel responsible for their health and see it as an important purpose [110,143]. Furthermore, the lack of awareness and knowledge of mHealth apps may negatively affect patients’ intentions to use them [58,60,102,127], especially with the vast number of apps available, which makes it difficult for patients to choose the one that suits them most [78,135].

### Social Influence and Cultural Factors

Several scholars argued that patients are often subject to the social influence surrounding them when making their health technology decisions [78,129,131,140,149,150], such as the presence or absence of caregivers who can encourage and support them in using the apps [70,107,108,115,128,132, 151,152], particularly in the case of people with less technology experience or those surrounded by a social circle that lacks technology experience [95,110,142]. Interestingly, the presence of strong social support and people who constantly care for the patient may sometimes discourage adoption as the person gets enough help from their caregiver and deems mHealth unnecessary [102]. It is also worth noting that social influence was not limited to the patient’s personal social network but also to the care team’s endorsement [81,153], input and support from other fellow patients who had undergone similar experiences through online communities and forums [104,113,118,154-157], or membership of a patient association [57]. Khalemsky et al [67] pointed out that this factor may also depend on the level of a person’s emotional autonomy, especially in the case of sick children and their relationship with their parents. In other contexts, researchers found no impact of social influence on adoption decisions [36].

Language barriers such as lack of language options in the tools may hinder adoption and compromise user experience [61,106,110,158], especially in the case of patients with low literacy [159,160]. This also applies to tools that use a complicated medical or technical language that is not easy for the patient to understand [78,158]. Conversely, Spooner et al [44] argued that the brevity and accessibility of some forms of mHealth tools, such as those using text messaging, may help overcome language barriers as they require less fluency compared with in-person or phone communication. Culture may also be an influencing factor, accounting for cultural nuances and tailoring the content to specific cultural beliefs and attitudes may foster adoption [95,118,121]. Gender issues in some cultural contexts may be a challenge; Duclos et al [115], for example, explains how male dominance may compromise mHealth implementation in some countries, as husbands prevent their wives from owning or using a phone.

### Technical and Material Factors

It is no surprise that technical factors related to mHealth tool features and capabilities also played a central role in adoption. Factors such as usefulness and ease of use are crucial for patient acceptance, as well as user experience and personalization, data-related factors, monetary factors such as cost and funding, and technical factors including access to technology and technical challenges.

### Usefulness

Perceived benefit and performance expectancy were among the key factors affecting patient acceptance of health care technologies, indicating that user adoption has much to do with the tool’s performance [40,76,78,81,92,94,101,110,114,129, 134,140,142,146,147,150,156,161,162], especially if they find it more useful compared with their current methods [87,106,117,141,157]. This perceived usefulness is not always related to the disease itself, but may also extend to other benefits such as better relaxation, an enhanced quality of sleep, or a sense of achievement [100,107,163]. In this context, it is important to note that a good understanding of the tool’s purpose and how it aims to help the patients may lead to higher adoption [60,132,137,164]. Furthermore, evidence of effectiveness may also encourage patients to start using the apps [43]. Similarly, lack of functionality or information [154] and lack of necessity or suitability [79,102,127,165,166] may lead to the tool’s abandonment. Surprisingly, Koivumäki et al [167] reported that their study found no impact of a tool’s performance on its adoption, contrary to most other studies.

Convenience and better access to care are typically facilitators to adoption [38,70,75,107,111,120,130,135,167,168], as mHealth tools may help save time and the cost of frequent clinic visits [77,105,128,137,151,169], are more flexible and may ﬁt better in the patients’ schedule [61,92,115,170], and immediate access to care may also be convenient, especially when it is not easy to reach a physician on weekends or in the evening, for example [79,127,141,144]. Some studies specified that longer travel times [34,65,96,103,116] and difficulties accessing traditional care services are similarly positively related to health app adoption [54,74,99,113,152]. Conversely, Kemp et al [119] reported that travel distance does not have a significant impact on adoption decisions.

Communication between patients and their care team and whether it is positively or negatively affected by the use of mHealth apps also affects patient acceptance [70,75,152,160,171]. Several studies have reported that mHealth may positively affect communications [128,130,131,136,141, 154,171], for instance, by enabling a quicker and easier exchange with their care team [60,61,79,80,98,104,113, 116,118], and hence foster adoption. In contrast, other scholars concluded that in some contexts, users may perceive a negative impact on their communication with their care team [69,92,111,115,173], as it is less personal [52,107,146,165, 168,169], leading to lower acceptance and adoption. It is worth noting that some studies have pointed out the importance of combining web-based and offline communication to encourage adoption, suggesting that mHealth should complement traditional care and not replace it [79,137].

Health education was perceived as a facilitator of mHealth adoption in all included studies [88,105,107,113,116,118, 147,152], and the educational and informative content in the apps may address knowledge gaps, raise disease awareness, and encourage healthier behaviors. Such benefits may encourage patients to accept these tools as they help them better understand their medication and possible drug interactions [40,42,79,104,130], their symptoms [101,136], and their specific condition [38,53,80,102,141,149,154,155,170,174,175], and hence achieve better health results [140,145,157].

Self-management is another factor that is predominantly perceived as a facilitator [104], helping patients be more proactive in coping with their condition [43,164], more conscious of their health condition and behaviors [60,117,141,145,157], more engaged in self-care [61,70,75,79,80,101,109,113,118,139,140,152,163], and feeling more secure and confident in managing their disease [120,131,136,149,161]. This particularly applies in the case of newly diagnosed patients, as it may help them build and adopt new habits to better manage their condition [144]. Woo and Dowding [102] found that patients who have been successfully managing their condition using traditional methods for a long time may be reluctant to adopt mHealth tools as they may fail to see their value. Conversely, Fairbrother et al [175] reported that patients may not engage in self-management as they perceive this to be the responsibility of their care team, so they may choose to adopt mHealth to enable their care teams to better monitor them but not to engage in proactive management of their own condition.

Several studies have reported that mHealth adoption may improve health outcomes [42,55,114,149]. Patients who perceive potential health benefits such as better health effects and enhanced health behaviors resulting from the use of these apps are more likely to adopt them [49,58,98,107,117,138,140,141]. Similarly, tools that target a better overall quality of life that go beyond solely focusing on the disease or health condition are usually highly appreciated and may have better chances of being accepted by patients [70,103,135,176].

Continuous monitoring may encourage adoption as it increases patients’ feeling of safety because their care team constantly monitors them [77,79,80,120,170,174], allowing for treatment optimization and better control of the condition [107,145], and a clearer overview of patients’ development for better follow-up [104,155]. Early detection of symptoms and health care issues is another benefit closely related to monitoring and may foster adoption, as the tools allow the care team to stay in the loop between clinic visits and intervene in case of symptom deterioration [40,80,87,101,107,144].

Seeking a better quality of care as an outcome of mHealth adoption may motivate user acceptance, several studies reported on quality improvement and better continuity of care [42,118,168-170,172], streamlining the processes of follow-up and care management [61,113,163], enhanced documentation and evidence-based health decisions [174], and a more holistic and individualized care approach [79,135], as potential facilitators. Personalized feedback is a closely related factor that may also enhance the overall quality of care and facilitate adoption as it enables a more patient-centric approach tailored to each patient’s individual needs [95,101,147,170].

### Ease of Use

Ease of use is one of the leading factors affecting mHealth adoption [70,104,107,117,150,160,161,163,177], patients would typically abandon tools that are complex or require a lot of effort [55,56,59,60,97,120,135,142,162,165,175], especially when they are already burdened by their condition [77,129]. In contrast, easy-to-use technologies that do not overburden patients have higher odds of being accepted and adopted [36,38,49,57,58,76,78,94,101,102,112,114,130,131,134,136,139,149,157,170]. Some studies have suggested that users’ perception of ease of use may be enhanced with good training material that shows the user how to optimize their use of these technologies [72,110,132,147], and by applying a more participatory approach to design that ensures the inclusion of patients in the development of tools [81,95].

### User Experience

Usability was often mentioned in the included study, especially with the multitude of tools available to patients to choose from; they would most likely adopt tools that give them the best user experience [40,60,107,129,137,139,173]. Elements such as app appearance and attractiveness, including font size, navigation, layout, colors, text length, automated features, and interactive design, may play a role in the adoption decision [78,130,135,146,152,154,157,162,178]. Some studies have pointed out that design factors such as font size, color brightness, and screen size may play a particularly important role with more senior users and therefore must be tailored to their cognitive and physical capabilities [101,110,136].

Personalization has been specifically mentioned in several studies; for instance, the inability to personalize the app according to their specific needs (eg, diagnosis, symptoms, medication, stage of treatment) may lead to lower adoption or even abandonment of the tool [98,146,162,164]. Patients often prefer to be able to adjust the tools to their specific needs [101,104,107,118,130,138,154,157,160]; for instance, the timing of prompts and frequency of reminders [164], adjusting the app to their preferred goals and activities [147,165], and adjusting visual features such as colors and text size [78,118]. It is worth noting that Zhang et al [133] pointed out that patients’ desire to have more personalized solutions may be related to a decrease in their privacy concerns.

### Data-Related Factors

Privacy and security are without a doubt very important factors; they were mostly perceived as a concern and a barrier to adoption, with many studies reporting on the importance patients put on the protection of their personal health information [38,40,43,52,55,61,85,87,97,101,108,128,130,137,139,168,169,175], typically requesting to know who will have access to their data and how the data will be protected against cybercrime [77,104,107,116-118,129,141,146,147,152,160,165], and sometimes voicing concerns or demanding control on whom to access their information, including other family members [58,155,157].

Conversely, some studies found that privacy may also facilitate adoption when patients perceive the apps to be secure and to offer a private way of sharing their health data [57,167], especially with users who already practice high privacy measures such as locking their phones with strong passwords [96]. Interestingly, van Heerden et al [174] pointed out that clinicians and patients are already using their smartphones to communicate and exchange information, which makes mHealth tools a more private and secure option compared with generic communication apps.

Other studies reported mixed outcomes regarding data privacy and security, expressing that not all participants perceived this factor as a barrier or as a facilitator but recognized both the advantages and the threats that it brings and highlighted the importance of securing the data [78,92,154,172]. For instance, Amann et al [144] explained that although some participants expressed concerns about data privacy, they also acknowledged that it is necessary to obtain the support they need through the app. Bauer et al [164] reported that although patients felt reassured knowing that their care team could access data about their symptoms through the app, they were simultaneously concerned about who else could have access to these data. Lupiáñez-Villanueva et al [93] concluded that patients do not have the same sensitivity to data privacy, and that their level of sensitivity may differ from one context to another. Interestingly, their study found that even users who are quite concerned about privacy are not necessarily willing to pay for it but rather would prefer their data to be protected by legal requirements [93]. Nonetheless, a few studies reported that they found no, or very minimal, impact of data privacy concerns on the adoption decision [69,94,120].

Quality, credibility, and reliability of the data available through mHealth tools may also play a role in the adoption decision [107,109,127,130,179]. The credibility of the information on the tool from the patients’ perspective often increases when it is provided or endorsed by trusted sources [40,58,78,93,110,116,154,175], reassurance that the information on the app is up-to-date to ensure its accuracy [144], and scientific evidence that warrants the app’s safety and reliability [57,59,101,113,118]. Relevance and appropriateness of the information offered by the app may also affect patient acceptance; content that is appropriate for users may foster adoption [118,146], whereas information that is not relevant, inappropriate, or not tailored to patients’ needs may discourage adoption [117,137,162]. For instance, Connor et al [154] explained that even an inappropriate tone, such as pushing too many tips through an app, could lead users to abandon it, especially if they are very sick.

### Monetary Factors

Monetary factors such as app costs and lack of reimbursement were mostly perceived as barriers to adoption [61,91,131,169]. Several researchers have reported that patients may not be ready to pay for health apps or choose to pay only for the features that they find crucial for their perceived health benefits [78,104,106]. Hidden costs generated through extra data use were also mentioned as a potential barrier to adoption [55,58,97,118,152], which is particularly relevant in specific socioeconomic contexts where prepaid mobile services are the norm, and an overuse of the data package may result in service discontinuity [166]. Additional costs resulting from the patient’s need to buy new technology to facilitate mHealth use may also deter adoption [52]. Conversely, mHealth affordability was reported as one of the facilitating factors in other studies [111,116,167], and it could even help save costs, mostly by saving travel time and expenses [43,80,105,113,151,172]. Interestingly, other researchers have reported no impact of mHealth costs on patients’ intentions to use mHealth [81,129,142]. Other scholars reported mixed or inconclusive results, stating that some users may be more cost-sensitive than others [42,65,92,93,126,168], for instance, younger users may be less willing to pay for health apps [65,168].

### Technical Factors

Technical issues were frequently cited as a barrier, with issues such as technology failure, insufficient phone storage, battery drain, syncing, and technical difficulties creating frustration and discouraging adoption [37,68,70,77,80,100,114,118-120, 130-132,135,137,138,141,151,178]. Poor technology infrastructure, including connectivity, network availability, and Wi-Fi issues [65,77,78,102,112,116,118,120,132,148,152, 155,158,166] as well as log-in difficulties [78,152,170] were also prevalent in the included studies. Access to technology is another important technical factor that should not be overlooked. Several studies have reported that the lack of patient access to technologies such as smartphones, computers, or specific apps [34,43,66,85,92,100,103,107,108,115,128,132,166], or lack of internet access [37,52,69,95,127], especially among older patients [135,157] could be barriers to mHealth adoption.

Training emerged in several studies as a particularly important factor for adoption given the disparity of technical skills among patients, especially in the older age groups and users with low levels of education [70,79,80,100,108,116,117,131,157,162]. The lack of such training may be a major concern and a real barrier to adoption [61,171,174]. Furthermore, technical support has often been cited as a facilitator to patient adoption if it is available and efficient in helping users overcome their technical issues [70,102,131], but it could also be a barrier if it is not adequate, leading users to abandon the tools when they do not feel supported when they face technical difficulties [55,117].

### Health-Related Factors

Health- and health care–related factors were equally central in the included studies. Elements such as the specific disease a patient has, the severity of their health condition, their health behavior, health consciousness and literacy, the relation of the mHealth tool to other therapies, and the role that the care team plays may affect a patient’s willingness to use mHealth tools. The patients’ disease and health condition may affect their decision to adopt mHealth. The severity of symptoms and complexity of the health condition were prevalent factors in the included studies; however, there were mixed results on whether they were a barrier or a facilitator. It is worth noting that the studies that established that more severe disease could be a barrier to adoption were about twice the number of studies that found it to be a facilitator.

Several researchers have reported that their studies found that patients with low baseline health, worse baseline clinical disease activity, higher prevalence of chronic conditions, high level of comorbidity, higher levels of pain and fatigue, higher frequency of hospital readmission, and those who were hospitalized or in the end-of-life phase were less likely to use the apps [52,64-66,68,73,76,80,107,112,114,115,119,148,159,161]. This could sometimes be explained by the closer follow-up usually needed by patients with a worse condition, resulting in a reduced need for mHealth [57]. In some specific cases, such as mental health disease, patients having a depressive episode, or those with more depressive symptoms, for example, may experience a sense of hopelessness that makes them disengaged in many aspects of life, including health care apps [104,121]; similarly, patients with severe psychotic symptoms may have an exaggerated sense of fear of the potential surveillance resulting from remote monitoring apps [117].

Conversely, other studies found that patients who are more affected by their disease or health state may be more motivated to use mHealth to manage their condition better [39,41,49,83,89,98,132]. For instance, Ross et al [138] reported that patients with higher pain ratings had a higher adoption rate, most likely because their perceived benefit from the app is higher compared with those who have pain levels under control. Similarly, Runz-Jørgensen et al [79] explained that patients with a higher burden of illness placed a higher value on the benefits that they could obtain from mHealth. Interestingly, 3 studies concluded that disease and health condition did not have a significant impact on patients’ decision to use mHealth [35,48,100].

Some studies have also reported that the disease type may be a factor that affects patients’ intentions to adopt health apps [83,90,91,120]. For instance, health care technologies seem to be more accepted among mental health patients compared with other conditions [50,74]. Bauer et al [86] reported that mHealth use appears to be more common among primary care patients compared with those with chronic conditions; however, they rationalized that this pattern may be explained by other factors such as older age in chronic disease patients and not their health condition as such. Torrent-Sellens et al [83] affirmed that the presence of specific types of diseases such as diabetes, stroke or cerebral hemorrhage, cancer, and cataract may increase the odds of mHealth adoption. Patients’ perception of the risk or health threat caused by their disease could also play a role in their adoption decision. A higher perception of risk or health threat may positively affect the adoption of health care technologies [36,110,123,129]. In addition, a higher stigma perception of the disease, such as in the case of HIV, may foster mHealth adoption [92].

The role of the care team is also central for adoption. It has mostly been reported as a facilitator, especially when the health app has been recommended by the health care provider [59,60,70,78,81,93,97,180], when patients notice how their care team responds to the data they feed into the apps and integrate it into their care [136,164], and when the care team offers coaching and support toward patients’ self-management [132,134,143]. However, Gupta et al [85] warned that clinicians should be careful not to overdo the reminders to use the tools, especially with patients with high disease burden, such as patients with cancer, to avoid overwhelming them. Several studies concluded that the care team could be a barrier to adoption if they lacked the necessary skills [107,168], if they did not proactively support mHealth use [44,62,101,118,154], or if they did not monitor the information that patients submit to the apps [80].

Interestingly, some studies reported mixed results; for example, clinician engagement and support of mHealth use may depend on their medical specialty, with specialists more engaged than general practitioners in health care app use, perhaps because of their higher involvement in shared decision-making and clinician-patient communication [135,153]. It may also be confusing to patients when the care team encourages them to use the technology, but then fails to actively monitor the data they feed into the apps, which eventually leads to app abandonment even if the user initially agrees to adopt the tool [145]. Magnol et al [57] explained that although physician’s recommendation could initially foster mHealth adoption, their potential lack of information on the range of available apps may also be a limitation.

Health consciousness and literacy could play a role in patients’ adoption of health care technologies [40,49,51,65,72,98,107,121, 134,159], as people with higher levels of health consciousness and literacy are typically more cognizant of their health issues and behaviors [93,110,142,156]. However, some studies have concluded that health literacy is not necessarily a significant predictor of mHealth use [73,81,86]. Health behavior is another factor with mixed results. Studies have reported that patients with a positive baseline health behavior, such as better medication adherence rate or a higher physical activity level, were more likely to adopt the tools [41,49,75,106]. Conversely, other researchers found that users with poorer baseline health behavior, such as a lower treatment adherence rate, felt a higher need for the app and used it more frequently [67]. Although Meyerowitz-Katz et al [98] reported a low adoption rate among those who were already healthy eaters before the initiation of mHealth use; Browning et al [48] found no correlation between baseline health behavior and mHealth use in their research.

Relation to other therapies and how the app fits into the overall patient journey and treatment path could play a role in the adoption decision. Several researchers have pointed out that although patients may appreciate the benefits they receive from mHealth, they still perceive it as a complement rather than a replacement for other components and modes of treatment [100]. When mHealth apps are used in isolation from other parts of the treatment and are not integrated into the overall patient journey, adoption rates may suffer [112-114,141,162]. Similarly, it is very important to consider any underlying comorbidities that the patients may have from before using mHealth to ensure a holistic understanding of the data they submit in the apps [80]. The type and burden of other medications may also play a role; for instance, the high burden of cancer treatment can be overwhelming, preventing patients from using an additional tool such as a health app [85]. Furthermore, patients who take multiple long-term medications and those who engage in multiple interventions may be less likely to adopt these tools [52,98]. Jemere et al [96] found that study participants who took medication in the form of a pill were over 3 times more likely to adopt mHealth compared with those who took medication in the form of an injection. It is also worth noting that some studies found that patients who have easy access to satisfactory care services or those who need frequent hospital follow-ups or hospitalization may have a lower mHealth adoption rate because they are often in direct contact with their care team [54,57,102,179].

Insurance status and its impact on mHealth adoption was inconclusive in the included studies. For instance, being publicly insured has been reported as a facilitator in a study by Pierce et al [71] but as a barrier in a study by Warinner et al [90]. Similarly, Anosike et al [126] found that some insured patients are less likely to use mHealth tools if they are not covered by their insurance, and Pierce et al [71] reported that privately insured patients are less likely to use these tools compared with Medicare, Medicaid, and self-pay patients. Others reported that patients who had commercial insurance or preferred provider organization insurance were more likely to use these services [37,82]. It is worth noting that adoption decisions related to patients’ insurance status may differ from one country to another depending on elements such as the legal requirements of minimum insurance cover and local policies on mHealth reimbursement.

## Discussion

### Practical Implications

This review builds on the growing body of research that investigates patients’ adoption of mHealth services and highlights the complexity of the factors affecting adoption, spanning personal, social, technical, organizational, and health care aspects. This implies that to achieve successful adoption and implementation of these tools, the different players in the health care landscape need to work together to overcome the barriers and harness the potential benefits of novel technologies in health care. Our findings show that mHealth developers and technology providers alone are not likely to achieve success by focusing on creating tools that are technically superior; there are social, organizational, health care, and policy-related factors that must be considered, underlining the central role of care teams and health care policy in promoting adoption.

**Figure 7:**
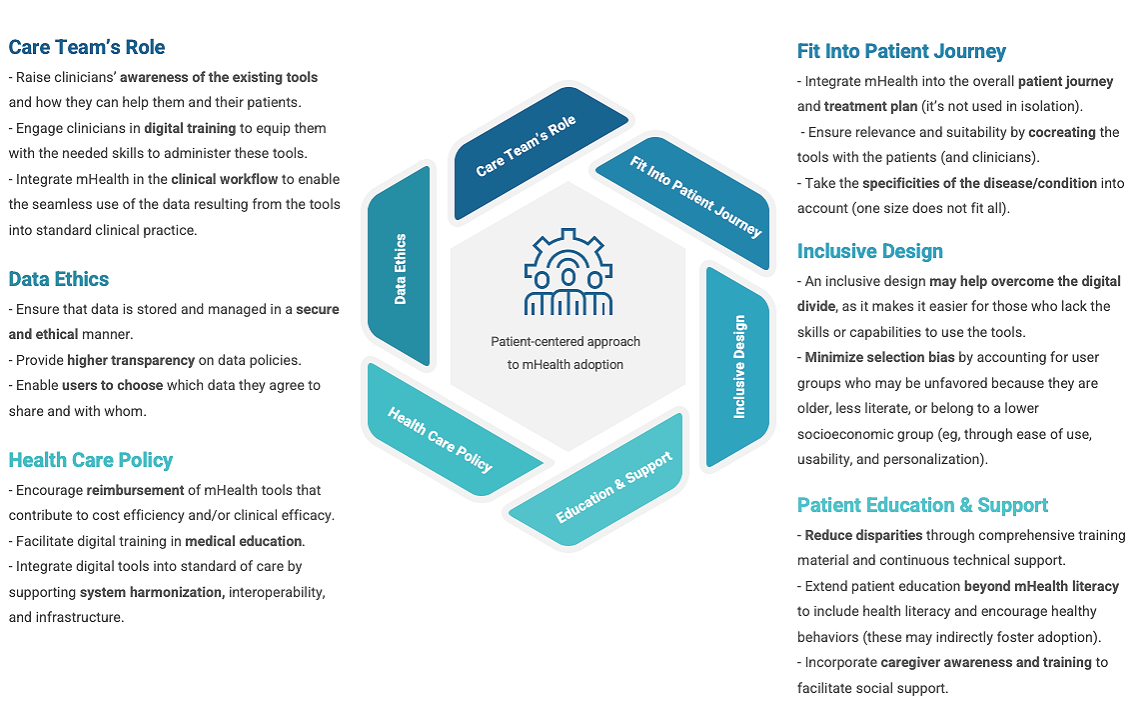
Recommendations for a patient-centered approach to mobile health (mHealth) adoption.

Although some factors may be very hard to influence (eg, intrinsic motivation or a person’s locus of control), others could be shifted. Figure 7 summarizes our recommendations for a more patient-centered approach to mHealth adoption, covering aspects that may help overcome some of the key barriers reported in this systematic review. This shift may be possible by ensuring the tools’ fit into the overall patient journey and treatment plan, emphasizing inclusive design, warranting comprehensive patient education and support, empowering and mobilizing clinicians and care teams, addressing ethical data management issues, and focusing on health care policies that may facilitate adoption.

Ensuring the tools’ *fit into the overall patient journey and treatment plan***,** based on the understanding that mHealth apps are not used in isolation, is crucial for sustainable adoption. Technology providers may opt to co-create the tools with patients (and clinicians) to ensure that they have taken their overall journey into account and established how their tool relates to other treatments that the patients are receiving, any comorbidities, and how their specific health condition may influence the way they use the technology [181]. Embedding users as equal partners in all phases of the development process may increase the usefulness, relevance, and appropriateness of the resulting tools, ensuring that they reflect the specificities of each disease and the overall context of the patients, increasing the odds of their adoption.

*Inclusive design* principles may help developers address the needs of the most vulnerable patient populations who may not be engaging with mHealth owing to their age or health-related physical and cognitive challenges, educational level, socioeconomic status, or their technological skills and experience. Numerous studies have concluded that many demographic factors are typically not the root cause for the lack of adoption per se, but rather other underlying causes were at play, mainly pointing back to a lack of skills and literacy that were typically correlated to more older patients, those with a lower level of education, or those belonging to lower socioeconomic classes. Designing for inclusivity does not ignore the unique features, environments, and cultural contexts of users. Research has shown that many aspects of the digital divide may be addressed through an inclusive design that incorporates cultural appropriateness, easy-to-understand lay language that does not need high literacy levels, and ease-of-use that does not require any sophisticated technical skills. For instance, a design that enables offline use may encourage patients in lower socioeconomic classes who are weary of the overuse of their data package to use the tool. Increasing the personal relevance of tools through personalization may also help address the varying needs of different users, allowing technology providers to cater to different patient populations that may vary in their level of skills, physical or mental capabilities, and literacy.

Another element that may help to reduce disparities in adoption is *patient education and support*. Comprehensive training materials and continuous technical support may assist some of the most unfavorable patient populations to benefit from these tools. Several studies have reported that the availability of training enables user groups that do not necessarily have the skills or literacy levels to acquire the knowledge that they need to use the tools more easily, especially when it increases their understanding of how the tool may help them improve their condition, step-by-step instructions on how it works, and knowing whom to contact in case of issues or questions. It is worth noting that extending patient education and awareness programs to go beyond mHealth literacy to include health literacy in general and encourage healthy behaviors may foster adoption, as research has shown that these factors may indirectly promote the tools’ uptake. Furthermore, given the important role of social influence, raising caregivers’ awareness may contribute to more successful adoption.

*Data ethics* is one of the most prominent factors in almost all health technology–related discussions, mostly as a barrier to adoption. Fostering patient adoption necessitates addressing their main fears and concerns by ensuring that their health data are stored and managed in a secure and ethical manner, providing higher transparency on data policies, and, whenever possible, enabling users to choose which data they agree to share and with whom.

*Care teams’ role* is central to patient adoption, as research shows that the endorsement of the clinician is a key facilitator of patient acceptance of the tools. However, lack of knowledge, skills, or active engagement with mHealth from the care team may discourage patient adoption. Therefore, raising clinicians’ awareness of the existing tools and how they can help them and their patients and engaging them in digital training to equip them with the necessary skills to administer these tools is central to success. Moreover, integrating mHealth in the clinical workflow to enable the seamless use of the data resulting from the tools in standard clinical practice is crucial, as previous studies have reported that patients would often abandon the tools if they feel that their care team is not actively engaging with the data that they feed on these apps.

Furthermore, recognizing potential barriers has essential *policy implications* for mHealth adoption to improve access to health care services and patient support. Encouraging the reimbursement of mHealth tools that contribute to cost efficiency and clinical efficacy may help overcome the cost-related barriers that were often reported in the studies. Facilitating digital training in medical education may help equip care teams with the necessary skills to implement and administer new technologies. Facilitating the integration of digital tools into the standard of care by supporting system harmonization, interoperability, and infrastructure may play a vital role in overcoming some of the key technical barriers that hinder adoption.

### Limitations and Recommendations for Future Research

Although this study contributes to the understanding of the factors affecting patients’ adoption of mHealth services, some limitations must be acknowledged. This review may not have included relevant studies that were not indexed in the searched databases, written in a language other than English, and gray literature searches that could have also allowed the identification of additional relevant insights. However, this study focused on peer-reviewed scientific papers.

In addition, this analysis only considered published studies, and no further contacts were made with the authors of the papers to obtain additional information or to validate our thematic analysis. Consequently, it is possible that other mHealth adoption factors may have been missed. Future reviews could include studies in other languages to gain a better grasp of any interregional or intercultural differences, and to have more studies in developed countries.

### Conclusions

This systematic literature review and narrative synthesis builds on and expands the growing body of literature investigating patients’ adoption of mHealth services. Our findings highlight the complexity of the factors affecting adoption, including personal, social, technical, organizational, and health care aspects. We recommend improving patient-centered approaches and taking a more holistic view of adoption factors beyond technical aspects by ensuring the tools’ fit into the overall patient journey and treatment plan. We emphasize the crucial role of inclusive design, which enables comprehensive patient education and support programs. Moreover, we stress the importance of empowering and mobilizing clinicians and care teams, addressing ethical data management issues, and focusing on health care policies that may facilitate adoption such as mHealth reimbursement.

## Appendix

Multimedia Appendix 1 Critical Appraisal Skills Program checklist.

Multimedia Appendix 2 Critical appraisal of the included studies.

Multimedia Appendix 3 Phases of thematic analysis based on Braun and Clarke [25].

Multimedia Appendix 4 Characteristics of included studies.

Multimedia Appendix 5 (A) Social and personal factors and their occurrence. (B) Technical and material factors and their occurrence. (C) Health-related factors and their occurrence.

## Abbreviations

mHealth: mobile health
PRISMA: Preferred Reporting Items for Systematic Reviews and Meta-Analyses
QCRI: Qatar Computing Research Institute
