# Supplementary material for: Sociotechnical Factors Affecting Patients’ Adoption of Mobile Health Tools: Systematic Literature Review and Narrative Synthesis
Source: JMIR Mhealth Uhealth. 2022 May 5;10(5):e36284. doi: 10.2196/36284 (PMC9121221; doi:10.2196/36284)
Supplement: Multimedia Appendix 4 [file mhealth_v10i5e36284_app4.pdf]

Appendix 4. Characteristics of included studies.

Study characteristic (n)		References
**Study Design**		
	Quantitative (73)	[34–44,46–49,51,52,55–57,62,63,65–67,69,72,74,76,77,81,83,84,86–94,96,97,99,108,109,119,120,123–126,128,129,132,133,139,142,143,149,150,156,159–163,166,169,167,179,180]
	Qualitative (43)	[53,59–61,75,78,79,100–107,111–117,130,135,137,140,141,144–147,151,152,154,155,157,158,165,168,172,174–176]
	Mixed methods (18)	[50,58,64,70,73,85,110,121,127,134,136,138,153,164,170,171,173,178]
	Systematic review (5)	[80,95,98,118,131]
	Others (8)	[45,54,68,71,82,122,148,177]
**Sample size**		
	Less than 10 (1)	[148]
	10-20 (21)	[79,80,98,101,102,105,106,113,115,131,135,137,140,145,151,152,154,155,162,164]
	21-40 (18)	[60,61,75,85,95,107,111,114,117,128,130,144,146,147,149,171,172,175]
	41-60 (10)	[59,103,104,108,112,118,136,158,169,173]
	61-80 (8)	[58,68,73,78,116,121,168,176]
	81-100 (8)	[45,67,100,143,153,157,170,177]
	More than 100 (80)	[34–44,46–57,62–66,69–72,74,76,77,81–84,86–94,96,97,99,109,110,119,120,122–127,129,132–134,138,139,141,142,150,156,160,161,163,166,167,178,180]
**Sample composition**		
	Patients (85)	[34–36,39–43,45–49,52–58,60,61,63,65,68–70,72–75,79–82,85–87,89,90,92,94,96,97,100,102,103,107,110,111,115,118–122,124–127,129,130,132,137–141,143,145,149–151,153,154,156,160,162–166,169,177,178]
	General public including patients (24)	[44,50,51,62,64,66,71,76,77,83,84,88,93,99,123,133,134,142,148,159,161,167,179,180]
	Patients plus caregivers (4)	[67,78,106,108]
	Patients plus HCPs (24)	[37,38,59,91,101,104,113,114,116,117,135,136,144,146,147,155,157,168,169–173,175,176]
	Patients plus HCPs plus others (e.g., researchers, policy makers…) (10)	[95,98,105,109,112,128,131,152,158,174]
**Disease area**		
	Diabetes and obesity (16)	[39,45,49,91,96,121,124,137,140,143,145,150,153,169,170,178]
	Cardiovascular disease and	[49,63,70,72,73,81,102,105,110,112,113,149,175]

	heart failure (13)	
	Mental health (13)	[35,41,100,104,107,117,141,142,146,157,165–168]
	Surgery (11)	[42,48,61,77,80,82,84,87,108,119,172]
	Oncology (10)	[37,53,56,85,90,106,125,135,162,177]
	Chronic diseases (9)	[46,74,95,97,98,126,136,161,163]
	Primary care (8)	[43,52,59,86,111,127,147,151]
	Neurology and neurosurgery (6)	[89,115,128,131,139,171]
	Others (28)	Blood pressure [36,120]; cirrhosis [132]; clinical trials [69]; collaborative care [164]; chronic obstructive pulmonary disease (COPD) [58,101,114]; HIV [92,173,174,176]; noncommunicable diseases (NCDs) [65]; ophthalmology [47]; pharmacovigilance and medication adherence [38,130]; physio, rehabilitation, and pain management [75,138]; rheumatology and immunology [40,57,67,68]; spine injury [144]; tuberculosis [116,158,160]; women's health [154,155]
**Location**		
	The United States (46)	[35,37,44,48,50,51,53,61–64,66,68,71,77,81,82,84–86,90,92,99,102,103,108,119–125,128,130,132,135,138,147,151,152,154,159,164,176,180]
	China (12)	[34,45,46,54,72,89,91,97,109,133,156,179]
	The United Kingdom (10)	[60,69,74,100,112,114,117,141,175,178]
	Canada (8)	[47,52,58,70,76,113,140,149]
	Australia (5)	[59,111,115,127,136]
	Germany (5)	[36,40,42,49,88]
	Singapore (5)	[43,56,106,110,153]
	Others (55)	Austria [75,165,169,170]; Bahrain [134]; Bangladesh [94,142]; Bolivia [65]; Burkina Faso[155]; Denmark [79,172]; Dominican Republic [168]; Ethiopia [96]; Finland [167]; France [57,104]; India [105,160,166]; Indonesia [158]; Israel [67]; Italy [143,162]; Jordan [129,162]; Netherlands [73,101,139,171]; Nigeria [126]; Norway [39,137]; Portugal [161]; South Africa [174]; South Korea [41]; Spain [146]; Switzerland [87,144]; Taiwan [145,163]; Uganda [173]; Multi country [38,78,80,83,93,95,98,107,116,118,131,150,157,177]
**Theoretical framework**		
	UTAUT[a] (12)	[36,60,70,78,102,110,129,142,149,150,161,167]
	TAM[b] (11)	[43,81,94,132–134,144,145,154,163,179]
	DOI[c] (2)	[127,157]
	Others (7)	Consolidated Framework for Implementation Research (CFIR) [103]; e-commerce acceptance model [176]; Elaboration-Likelihood Model [156]; The Method for Program Adaptation through Community Engagement (M-PACE) model [147]; The NASSS (non-adoption, abandonment, scale-up, spread, sustainability) framework [112]; The RE-AIM (reach, effectiveness, adoption, implementation, maintenance)

		framework [177]
