# Supplementary material for: Sociotechnical Factors Affecting Patients’ Adoption of Mobile Health Tools: Systematic Literature Review and Narrative Synthesis
Source: JMIR Mhealth Uhealth. 2022 May 5;10(5):e36284. doi: 10.2196/36284 (PMC9121221; doi:10.2196/36284)
Supplement: Multimedia Appendix 5 [file mhealth_v10i5e36284_app5.pdf]

Appendix 5.A. Social and Personal factors and their occurrence.

Factors (n)		References
**Moderating Factors**		
	Age (71)	Older age a barrier [34–67,70,86,88–91,93,97,98,118,121,122,124,125,132,150]; facilitator [68–71]; mixed results [77–85,95,104,105]; not a factor [72–76]
	Gender (34)	Being female a barrier [43,65,92,93]; facilitator [39,51,54,55,67,71,82,83,85–88]; mixed results [35,49,69,77,94,95,125]; not a factor [36,40,45,50,53,62,68,73,89–91]
	Education (34)	Lower level of education a barrier [35,42–44,46,49,55,57,64,65,72,84,89–93,96–98,125]; facilitator [45,88,99]; mixed results [39,41,51,56,78,82,83,95]; not a factor [68]
	Tech skills (30)	Lack of tech skills a barrier [43,52,61,62,69,72,74,77,79,80,88,100–105,108,113,114,127,131,132,172]; mixed results [58,64,66,106,107]; not a factor
	Tech experience (27)	Having a previous tech experience as a facilitator [38,40,46,48,49,54,56,78,83–85,92,96,97,102,108–118]; mixed results [127]
	Ethnicity (24)	Being non-white a barrier [35,62,64,66,71,84,119–122]; facilitator [43,44,51,77,124,125]; mixed results [56,69,95,123]; not a factor [50,53,86,90]
	Socioeconomic factors / Income (22)	Lower income a barrier [37,43,44,51,57,63,77,82–84,89–91,126,127]; mixed results [39,41,62,95]; not a factor [48,50,52]
	Geographic residence (9)	Rural residence a barrier [50,71,83,89]; facilitator [113]; mixed results [76,95]; not a factor [90,91]
	Marital status / living alone (7)	Being single or living alone a barrier [43,82,83,95,119]; facilitator [50]; not a factor [53]
**Personal Characteristics**		
	Attitudes / preferences (29)	[36,37,41,43,45,55,56,58,60,62,69,77,83,87,101–103,114,115,119,127–135]
	Psychological factors (17)	[67,69,73,79,80,85,101,107,112,115,118,136–140,178]
	Time constraint / distraction (16)	[55,69,80,91,100,110,112,115,116,118,120,132,137,138,140,141]
	Motivation (12)	[100,101,107,114,130,140,142–147]

	Locus of control / self-efficacy (7)	[80,110,140,143,167,178,180]
	Awareness (6)	[58,60,78,102,127,135]
	Habits (5)	[57,70,110,140,142]
**Cultural and Social Elements**		
	Social influence (30)	[36,57,67,70,78,81,95,102,104,107,108,110,113,115,118,128,129,131,132,140,142,149–157]
	Language (8)	[44,61,78,106,110,158–160]
	Culture (4)	[95,118,121,155]

Appendix 5.B. Technical and material factors and their occurrence.

Factors (n)	References
**Usefulness**	
Perceived benefits and performance expectancy (55)	[36,40,43,46,56,60,62,69,70,74,76,78,79,81,83,87,92,94,97,100–102,106,107,110,114,117,127,129,132–134,137,140–142,146–150,154–157,161–167,171,172,176]
Convenience and accessibility (40)	[34,38,43,54,59,61,65,70,74,75,77,79,92,96,99,103,105,107,111,113,115,116,119,120,127,128,130,131,135,137,141,144,151,152,154,157,168–171]
Communication (36)	[52,60,61,69,70,75,77,79,80,92,98,99,101,104,107,111,113,115,116,118,128,130,131,136,137,141,146,152,154,160,165,169,170,172–174]
Health education (33)	[38,40,42,53,58,77,79,80,88,101,102,104,105,107,113,116,118,130,136,140,141,144–147,149,152,154,155,157,171,175,176]
Self-management (31)	[43,60,61,70,75,79,80,98,101,102,104,109,113,117,118,120,131,136,139–141,144,145,149,152,157,161,163,164,171,175]
Quality of care (12)	[42,61,79,113,118,135,163,169–171,173,175]
Health benefits (12)	[42,49,55,58,98,107,114,117,138,140,141,149]
Monitoring (11)	[77,79,80,97,104,107,120,145,155,171,175]
Early detection of symptoms (6)	[40,80,87,101,107,144]
Personalized feedback (5)	[95,101,147,171,177]
Life quality and well-being (4)	[70,103,129,135]
**Ease of Use (54)**	[36,38,49,55–60,70,72,76–78,81,91,94,95,97,101,102,104,107,110,112,114,116,117,120,129–132,134–139,142,146,148–150,157,160–163,165,167,171,176,179]
**Technical Factors**	
Technical issues (43)	Technical failure or difficulties [37,52,58,68–70,77,80,100,114,118–120,130–132,135,137,138,141,145,148,151,155,175,178]; infrastructure [65,77,78,102,112,116,118,120,132,148,152,155,158,166]; login issues [78,152,171]
Access to technology (20)	[34,37,43,52,66,69,85,92,95,100,103,107,108,115,127,128,132,135,157,166]
Training (13)	[61,70,79,80,100,108,116,117,131,157,162,172,175]
Tech support (5)	[55,70,102,117,131]
**Monetary Factors (35)**	Monetary and cost aspects a barrier [52,55,58,61,78,91,97,101,104,106,107,118,130,131,152,166,1

		70]; facilitator [43,80,105,111,113,116,151,168,173]; mixed results [42,65,92,93,126,169]; not a factor [81,129,142]
**Data related**		
	Privacy and security (51)	Privacy and security a barrier [38,40,43,52,55,58,61,77,85,87,91,97,101,104,107,108,116–118,128–130,133,137,139,141,146,147,151,152,155,157,160,165,169,170,176]; facilitator [57,96,168,174]; mixed results [78,92,93,144,154,164,173]; not a factor [59,94,120]
	Quality, reliability, and credibility (20)	[40,57–59,78,93,94,101,107,109,110,113,116,118,127,130,144,154,176,179]
	Relevance (6)	[117,118,137,146,154,162]
**User Experience**		
	Usability (19)	[40,60,78,101,107,110,129,130,135–137,139,146,147,152,154,162,174,178]
	Personalization (17)	[78,98,101,104,107,118,130,133,138,146,147,154,157,160,162,164,165]

Appendix 5.C. Health related factors and their occurrence.

Factors (n)		References
**Disease or Health Condition**		
	Worse condition as a barrier (21)	[52,57,64–66,68,73,76,80,104,107,112,114,115,117,119,121,148,159,161,171]
	Worse condition as a facilitator (11)	[39,41,49,55,79,83,89,98,132,138,177]
	Disease type as a factor (7)	[50,74,83,86,90,91,120]
	Risk perception (5)	[36,92,110,123,129]
	Not a factor (3)	[35,48,100]
**Care team's role**		
	Facilitator (14)	[59,60,70,78,81,85,93,97,132,134,136,143,164,180]
	Barrier (8)	[44,62,80,101,107,118,154,169]
	Mixed results (4)	[72,125,142,175]
**Health consciousness and literacy (17)**		[40,49,51,65,72,73,81,86,93,98,107,110,121,134,142,156,159]
**Relation to other therapies (15)**		[52,54,57,80,85,96,98,100,102,112–114,141,162,179]
**Health behavior (7)**		[41,48,49,67,75,98,106]
**Insurance status (5)**		[37,71,82,90,126]
